# Non-senescent *Hydra* tolerates severe disturbances in the nuclear lamina

**DOI:** 10.18632/aging.101440

**Published:** 2018-05-10

**Authors:** Alexander Klimovich, Arvid Rehm, Jörg Wittlieb, Eva-Maria Herbst, Ricardo Benavente, Thomas C.G. Bosch

**Affiliations:** 1 Zoological Institute, Christian-Albrechts University of Kiel, Kiel D-24118, Germany; 2 Department of Cell and Developmental Biology, Biocenter, University of Würzburg, Würzburg D-97074, Germany

**Keywords:** non-senescence, stem cell, lamin, nuclear envelope, *Hydra*

## Abstract

The cnidarian *Hydra* is known for its unlimited lifespan and non-senescence, due to the indefinite self-renewal capacity of its stem cells. While proteins of the Lamin family are recognized as critical factors affecting senescence and longevity in human and mice, their putative role in the extreme longevity and non-senescence in long-living animals remains unknown. Here we analyze the role of a single lamin protein in non-senescence of *Hydra*. We demonstrate that proliferation of stem cells in *Hydra* is robust against the disturbance of Lamin expression and localization. While Lamin is indispensable for *Hydra*, the stem cells tolerate overexpression, downregulation and mislocalization of Lamin, and disturbances in the nuclear envelope structure. This extraordinary robustness may underlie the indefinite self-renewal capacity of stem cells and the non-senescence of *Hydra*. A relatively low complexity of the nuclear envelope architecture in basal Metazoa might allow for their extreme lifespans, while an increasing complexity of the nuclear architecture in bilaterians resulted in restricted lifespans.

## Introduction

**Figure 1:**
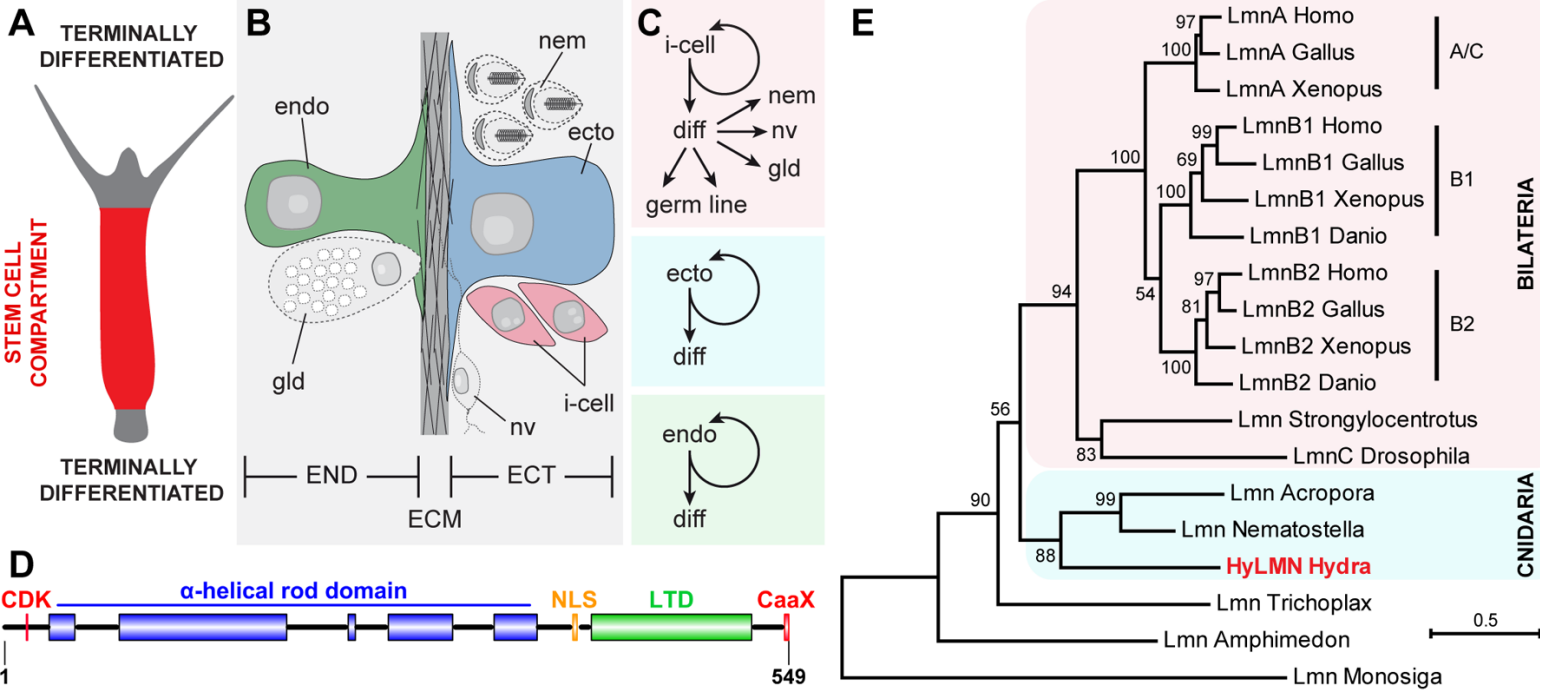
**Stem cells in *Hydra* express a single Lamin protein structurally similar to vertebrate B-type lamins.** (**A**) Stem cells continuously proliferate in the middle body column of *Hydra*, and undergo terminal differentiation at the upper and lower body column ends. (**B**) *Hydra* body is made of ectodermal (ECT) and endodermal (END) epithelial layers, separated by the extracellular matrix (ECM) called mesoglea. (**C**) Three stem cell lineages are present in *Hydra*. Interstitial stem cells (i-cell) differentiate (diff) into somatic cells - nematocytes (nem), nerve (nv) and gland (gd) cells, and germline cells. Ectodermal and endodermal lineages represent unipotent stem cells. (**D**) Single HyLMN protein in *Hydra* shows typical structural features of nuclear Lamins: N-terminal motif for phosphorylation by cyclin-dependent kinases (CDK, red), alfa-helical rod domain (blue), putative nuclear localization signal (NLS, orange), immunoglobulin-like lamin terminal domain (LTD, green) and a C-terminal CaaX-like motif (CaaX, red). (**E**) Phylogenetic tree of Lamin homologs clusters HyLMN protein among Lamins from other cnidarians at the basis of Metazoan tree. Maximum-likelihood phylogram rooted using the Lamin-like sequence from a choanoflagellate *Monosiga*. Numbers at nodes are bootstrap support values calculated by 1000 iterations. See Methods for sequence accession numbers.

The freshwater polyp *Hydra* belongs to the Cnidaria phylum, and represents a rare case of an animal with extreme longevity. It demonstrates unlimited clonal growth with no detectable signs of senescence, such as age-dependent increase in mortality or decrease in fertility, and thus is considered as non-senescent [1–4]. *Hydra* body is made of cells of three lineages, originating from unipotent ectodermal and endodermal epithelial stem cells, and from multipotent interstitial stem cells (Fig. 1A–C). In contrast to most other animals, stem cells in *Hydra* indefinitely maintain their self-renewal capacity, thus sustaining non-senescence and everlasting asexual growth [5,6]. While unlimited self-renewal capacity of the stem cells is long recognized fundamental for *Hydra*’s non-senescence, the underlying molecular mechanisms remain poorly understood. So far, the transcriptional factor FoxO was found as critical regulator of *Hydra* stem cell homeostasis and longevity, supporting the view that components of the insulin/insulin-like growth factor signaling pathways govern lifespan throughout the animal kingdom [7–10]. Several other transcriptional factors, such as POU domain-containing proteins and Myc family proteins, are supposed to contribute to the non-aging of *Hydra* and other cnidarians [11,12]. However, the putative effector molecules downstream from these transcriptional factors that might contribute to the sustained stem-cell activity and non-senescence in *Hydra* remain unclear.

Studies in bilaterian animals propose proteins of the Lamin family to be the major effector molecules involved in the age-related cellular senescence and, hence, in the genetic control of ageing and lifespan [13–16]. These highly conserved intermediate filament proteins form a complex network at the inner nuclear membrane, arrange the nuclear architecture and orchestrate multiple nuclear processes, such as DNA replication and repair, chromatin condensation and transcription [17–19]. Importantly, bilaterian cells are highly sensitive to the nuclear lamina disturbances. Decline in the expression level of Lamin B1 and increase of an aberrant Prelamin A isoform are associated with the age-dependent alterations in the nuclear lamina morphology and chromatin organization observed upon physiological ageing in mammals and invertebrates [20–23]. Furthermore, in human, mutations affecting the primary sequence of Lamin proteins, their expression level, or the activity of the Lamin-modifying enzymes cause nuclear envelope abnormalities that are linked to a wide range of diseases, collectively termed laminopathies. Individuals affected by such syndromes (*e.g.* Hutchinson-Gilford progeria, Nestor-Guillermo progeria syndrome) are characterized by accelerated ageing phenotype and gravely affected lifespan [24–26]. Finally, diverse experimental models corroborate the importance of the proper Lamin primary sequence, expression levels, and processing for the tissue homeostasis and lifespan [20,27–30]. Taken together, these findings point to the central role of Lamins in maintaining stem-cell activity and tissue homeostasis, as well as in controlling cellular and organismal senescence in model animals [14,17,31].

A homologue of vertebrate lamin B genes has been identified in *Hydra* [32], yet no efforts have been reported addressing the role of Lamin in cnidarian longevity. Here we present detailed analysis of the single *Hydra* lamin gene (*hyLMN*), its expression pattern, and distribution and function of its protein product (HyLMN). We show that *Hydra* stem cell proliferation displays an extraordinary robustness against the Lamin disturbance. This may play a critical role in the unlimited self-renewal capacity of *Hydra* stem cells and its non-senescence.

## RESULTS

### *hyLMN* gene has a highly conserved structure

To get first insights into the function of the single Lamin protein in *Hydra*, we analyzed the structure and phylogenetic position of the *hyLMN* gene. Direct cloning and sequencing of the *hyLMN* cDNA and analysis of the available *Hydra* genomic database revealed that *hyLMN* gene spans 27,165 bp in the genome, and is made up of 10 exons separated by 9 introns (Suppl. Fig. 1). Remarkably, the same number of exons and similar positions of exon-intron junctions are found in *lamin* genes of bilaterian animals, including humans [33]. The mature *hyLMN* mRNA includes an ORF of 1,647 bp, coding for a 549 amino acid long protein with predicted molecular weight of 63.9 kDa (Suppl. Fig. 1). *In silico* analysis of the deduced HyLMN protein sequence revealed a presence of all the features, typical for Lamins of invertebrates and type-B Lamins of vertebrate animals [34] (Fig. 1D, Suppl. Fig. 1). Phylogenetic analysis placed the HyLMN among Lamins from other cnidarians at the basis of Metazoan tree (Fig. 1E). This, together with the conserved exon-intron organization of *lamin* genes and conserved domain structure of the protein, is consistent with the view of a monophyletic origin of the Lamin gene family in Eumetazoa. Presumably, the first multicellular animals already had a nuclear envelope made of a single Lamin protein that had a structure further retained in the B-type Lamins of higher vertebrates [34,35].

**Figure 2:**
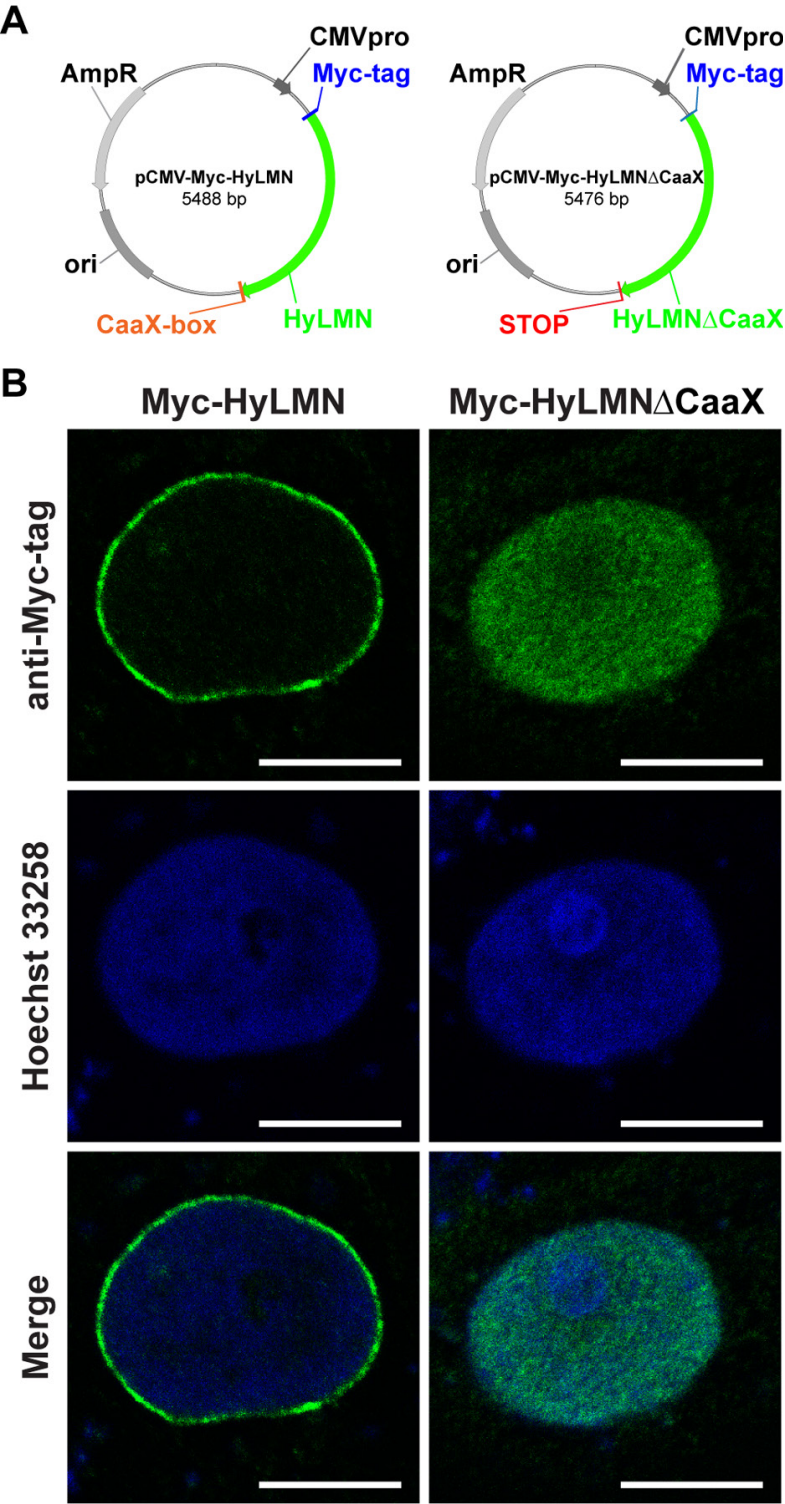
**Lamin protein from *Hydra* is recruited to the nuclear envelope of mammalian cells, if it contains an intact CaaX-box.** (**A**) Maps of two vectors used for the heterologous expression of *Hydra* HyLMN in COS-7 cells. (**B**) Transfection of COS-7 cells with a pCMV-Myc-Vector containing full CDS of the *hyLMN* results in the expression of Myc-tagged HyLMN protein (Myc-HyLMN) localized to the nuclear envelope. If a stop-codon is introduced into the *hyLMN* CDS resulting in the truncated HyLMN protein lacking the C-terminal CaaX-box (Myc-HyLMNΔCaaX), the protein is not integrated anymore into the nuclear envelope and accumulates in the nucleoplasm.

The high degree of HyLMN structural conservation points to a potential conservation in its function, and implies that in *Hydra* the HyLMN protein might be essential for the nuclear envelope formation, interaction with the nuclear membrane (via CaaX-box), and for the interplay with other nuclear proteins and the chromatin. We confirmed high functional conservation of HyLMN by expression of its full-length coding sequence in a heterologous mammalian system (Fig. 2). The overexpressed HyLMN fused to a Myc-tag showed clear distribution of the signal in the nuclear envelope of transfected COS-7 cells (Fig. 2), indicating that Lamin protein of *Hydra* successfully integrated into the lamina of mammalian cells. To investigate the functional importance of the C-terminal CaaX-motif [36] in HyLMN (Fig. 1D), we overexpressed a truncated version of HyLMN, where the last four amino acids comprising a CaaX-box were deleted, fused to Myc-tag (Fig. 2A). Deletion of the motif resulted in a homogeneous distribution of the tagged protein in the nucleoplasm (Fig. 2B). These results indicate a crucial role of the CaaX-motif for the protein’s localization and support the high degree of structural and functionalconservation of Lamin in *Hydra* and across the Eumetazoa.

### *hyLMN* gene is expressed in all stem cells of *Hydra*

**Figure 3:**
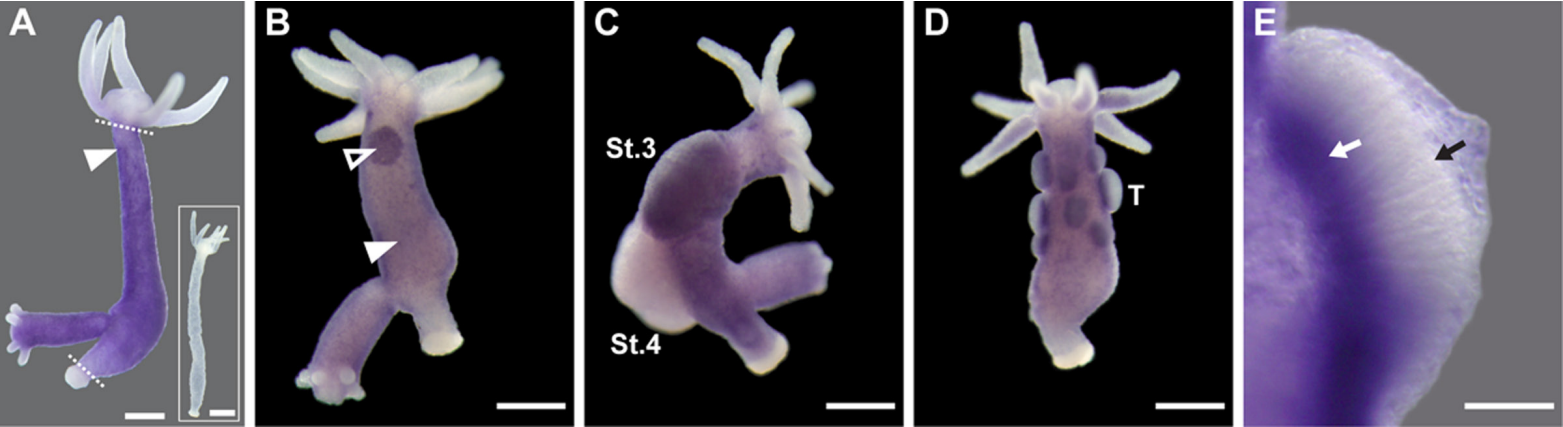
***HyLMN* is strongly expressed in the proliferating stem cells, but absent from the differentiated cells.** (**A**) Whole-mount *in situ* hybridization with a DIG-labeled antisense probe specific for *hyLMN* reveals that the expression of *hyLMN* mRNA is restricted to the stem cells compartment (marked out by two dashed lines), with highest signal observed in the interstitial cells (arrowhead). Hybridization with a sense probe (inset) gives no signal. (**B**) In a sexually-induced polyp, *hyLMN* mRNA is expressed at elevated levels in single interstitial cells (white arrowhead) and clusters of precursor cells (empty arrowhead) typical for early gonad formation. (**C**) In a female polyp, a strong *hyLMN* signal is detected in the early gonad on stage 3 of oogenesis (St.3) and absent in later oogenesis stages (St.4). (**D**-**E**) In a male polyp, strongest *hyLMN* expression is observed in the basis of testes (T on D; white arrow on E), where mitotically dividing precursor cells are located. In the apical zone of the testis no signal can be detected (black arrow on E), indicating an absence of the *hyLMN* transcript in post-meiotic cells. Scale bar: 300 μm (A-D), 50 μm (E).

To analyze the expression pattern of the *hyLMN* gene, we performed whole mount *in situ* hybridization with a specific digoxigenin-labeled RNA-probe. *HyLMN* transcript is present exclusively in the gastric region of a polyp, where stem cells are located, while the extremities (foot, hypostome and tentacles), where differentiated cells are found, are devoid of *hyLMN* mRNA (Fig. 1A, Fig. 3A). The strongest hybridization signal is detected in the sexually-induced polyps – in the spermatogonia zone at the testes basis in male polyps, and in early female gonads at the early oogenesis stages (Fig. 3B–E). Similarly to fully differentiated somatic cells, mature germline cells (sperms and oocytes) lack *hyLMN* mRNA (Fig. 3C–E). Taken together, these data suggest that the transcription of *hyLMN* is restricted to the intensively proliferating stem cells in *Hydra*.

**Figure 4:**
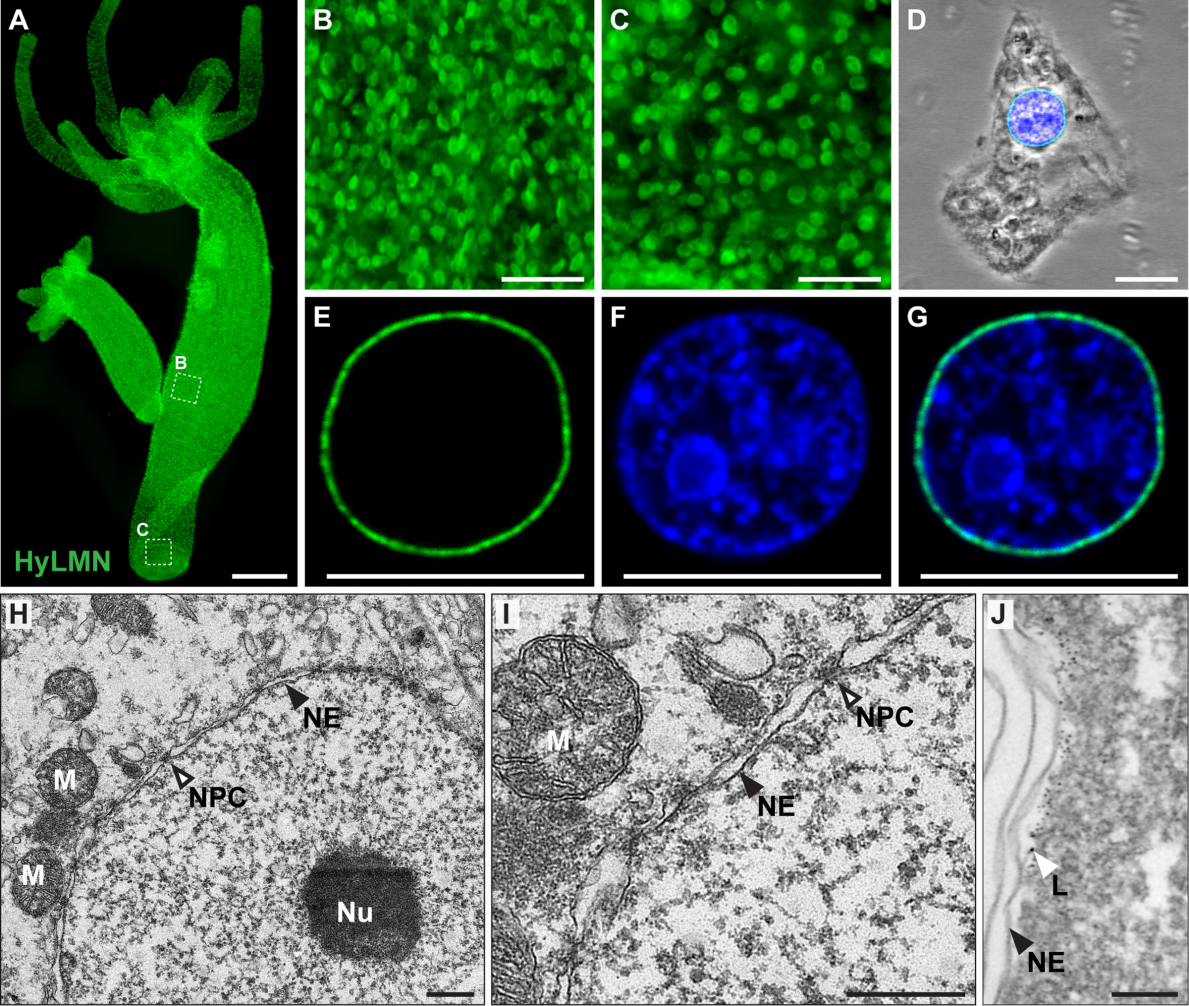
**HyLMN protein is present in the nuclei of every *Hydra* cell and forms a nuclear lamina.** (**A-C**) Immunostaining with anti-HyLMN antibodies reveals HyLMN protein in the nuclei of all cells in the polyp (A), including the stem-cell compartment (B) and the foot, made of differentiated cells (C). (**D-G**) HyLMN protein forms a thin layer - the lamina, surrounding the chromatin. Immunodetection of Lamin (green), DNA (blue), merged with phase contrast (on D). (**H, I**) Transmission electron microscopy reveals a typical organization of the nucleus in an epithelial cell of *Hydra*. Nuclear envelope (NE) consists of two membranes with incorporated nuclear pore complexes (NPC). The chromatin and a conspicuous nucleolus (Nu) are found within the nuclear envelope. Several mitochondria (M) are located close to the outer nuclear membrane. (**J**) Electron microscopy immunolocalization of the HyLMN protein shows that the lamina (L), labeled by the 6 nm gold particles, lays beneath the inner membrane of the nuclear envelope (NE). Scale bar: 300 μm (A), 50 μm (B-C), 10 μm (D-G), 500 nm (H-I), 100 nm (J).

To reveal the localization of HyLMN protein, we performed immunostaining with specific polyclonal antibodies raised against a 146 amino acid long peptide of HyLMN. Whole-mount immunofluorescent staining demonstrates that HyLMN is expressed in nearly every cell throughout the entire *Hydra* body: the epitope was detected in the nuclei of cells in the gastric region, in the hypostome and tentacles, and in the foot (Fig. 4A–C). Immunodetection of HyLMN on macerated cells showed that Lamin protein is strongly expressed in the stem cells of three cell lineages – the ectoderm, endoderm and in the interstitial stem cells, as well as in their differentiated progeny (Fig. 4D–G, Suppl. Fig. 2). Only in the late spermatocytes, spermatids and spermatozoa no HyLMN protein can be detected (Suppl. Fig. 3). Taken together, our expression analysis reveals that though *hyLMN* mRNA is produced only in the intensively proliferating stem cells, the HyLMN protein is present in virtually all *Hydra* cells.

### Dynamics of HyLMN in the nuclear envelope

**Figure 5:**
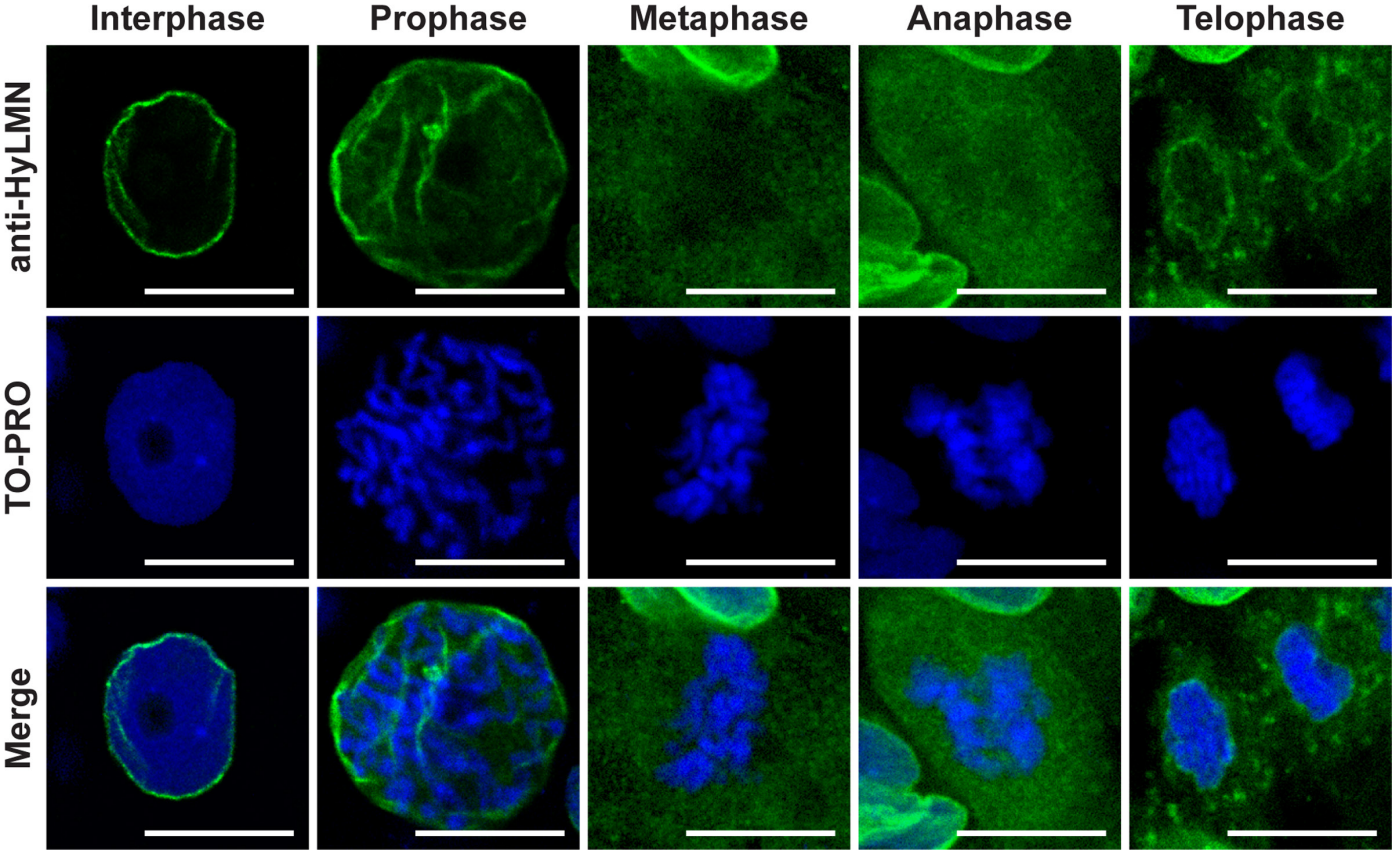
**HyLMN displays a dynamic distribution in the nucleus through the cell cycle.** A series of images from ectodermal epithelial cells at different phases of the mitotic cell cycle. In an interphase nucleus, HyLMN forms a thin rim around the chromatin - the lamina. In the prophase, with the onset of chromosome condensation, fragmentation of the nuclear lamina starts. In the metaphase and anaphase, when chromosomes are completely condensed, form a metaphase plate and are separated in two sets, HyLMN is redistributed to the cytoplasmic pool. In the telophase two separated sets of chromosomes are enclosed within new forming envelopes. From a homogeneous cytoplasmic pool HyLMN aggregates to form laminas of the daughter cells. Immunodetection of Lamin (anti-HyLMN, green), DNA (TO-PRO, blue), and merged image. Scale bar: 10 μm.

Immunochemical staining revealed, furthermore, a dynamic distribution of HyLMN in the cell along the cell cycle. In an interphase nucleus, HyLMN is localized to the inner site of the nuclear envelope and surrounds the chromatin (Fig. 4D–G, Fig.5, Suppl. Fig. 2). Consistently with these observations, immunogold staining revealed localization of HyLMN in the nucleus, in a close proximity to the inner membrane of the nuclear envelope (Fig. 4H–J). In the prophase of mitosis the nuclear lamina is fragmented, and most of the HyLMN protein is redistributed to the cytoplasmic pool in the metaphase and anaphase (Fig. 5). Finally, in the telophase the nuclear laminas of the daughter cells are assembled again (Fig. 5). This dynamic HyLMN distribution is observed in proliferating cells of all three *Hydra* stem cell lineages. Together, localization of HyLMN protein to the nuclear envelope and its dynamic distribution in *Hydra* cells on different cell cycle stages reflect the behavior reported for Lamin proteins in invertebrates and vertebrates [37], highlighting functional conservation of the protein.

### Overexpression of HyLMN does not affect stem cell activity

**Figure 6:**
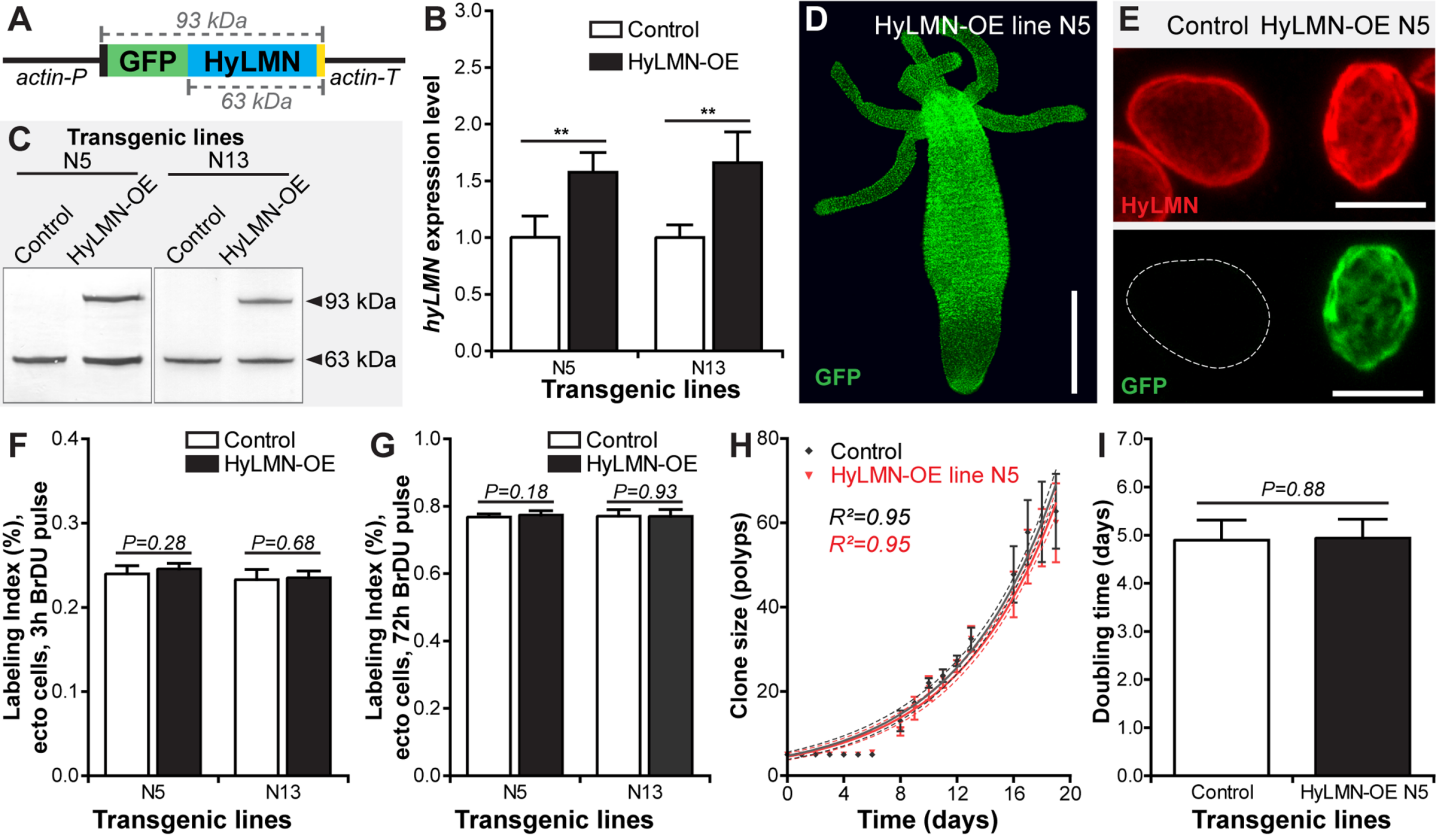
**Overexpression of HyLMN does not affect stem cell activity.** (**A**) Genetic construct used for *hyLMN* overexpression. Actin promotor (actin-P, 1420 bp) drives the expression of the GFP-HyLMN fusion protein (806 a.a., 93 kDa) with an intact C-terminal CaaX box (yellow). Actin terminator (actin-T, 701 bp) flanks the sequence. (**B**) *hyLMN* mRNA expression levels in two ectodermal HyLMN-OE lines (N5 and N13) and respective controls, analyzed by qRT-PCR (*n*=6, mean±S.D.). Asterisks indicate significant changes in expression levels (Mann-Whitney test); P values: N5 line = 0.002, N13 line = 0.002. (**C**) Western-Blot with anti-HyLMN antibodies confirms expression of the fusion protein GFP-HyLMN (93 kDa) along with the endogeneous HyLMN (63 kDa) in two transgenic lines. In control lines, only endogenous HyLMN is detected. (**D**) A polyp overexpressing GFP-HyLMN in all ectodermal cells, stained with anti-GFP antibodies. Scale bar: 500 μm.(**E**) Overexpression of HyLMN results in an uneven distribution of the protein in the nuclear lamina, evidenced by the immunostaining with anti-HyLMN and anti-GFP antibodies. Scale bar: 10 μm.(**F**) BrdU-labeling index of the ectodermal epithelial cells in HyLMN-OE (N5 and N13 lines) and control polyps after 3 h exposure to BrdU (N5 *n*=10, N13 *n*=13, 513.6±51.3 cells per replicate, mean±S.D.). (**G**) BrdU-labeling index of ectodermal epithelial cells in HyLMN-OE (N5 and N13 lines) and control polyps after 72 h exposure to BrdU (N5 *n*=15, N13 *n*=15, 514.9±20.4 cells per replicate, mean±S.D.). (**H**) Growth curves for the HyLMN-OE line N5 and control polyps (*n*=4 replicates, each five polyps on day 0; mean±S.D., linear regression lines with 95% CI corridors and goodness of fit R^2^). (**I**) Population doubling time (mean±95% CI) for HyLMN-OE line N5 and control polyps derived from the plot on H.

To address the function of the HyLMN protein in *Hydra*, we implemented transgenesis technology [5], and first overexpressed the entire HyLMN coding sequence fused to GFP under a ubiquitous promotor (Fig. 6A). Two transgenic lines (N5 and N13) revealed 1.5-fold up-regulation of *hyLMN* mRNA expression (Fig. 6B) in total polyps compared to the corresponding controls (so called “empty” polyps). However, since in the both strains the transgene was integrated only into the ectodermal cell lineage, while cells of other two (endodermal and interstitial) lineages remained intact, the up-regulation level in the transgenic cell lineage is likely even higher, about 5-fold. Western-blot analysis confirmed successful expression of the fusion protein. In addition to a band of a molecular weight of 63 kDa present in protein extracts from both, control and transgenic *Hydras,* and corresponding to the endogenous HyLMN protein, a band with an apparent molecular weight of 93 kDa was detected in the transgenic polyps (Fig. 6C). This size corresponds to the expected molecular weight predicted by summing up the size of HyLMN (63 kDa, Suppl. Fig. 1) and GFP (30 kDa, Fig. 6A). These observations also provide an additional evidence for the high specificity of the anti-HyLMN antibody. Overexpressed fusion protein is present in all ectodermal cells of transgenic polyps (Fig. 6D) and localized to the nuclear envelope, as anticipated since the C-terminal CaaX-box is present and not affected by fusion to GFP. Remarkably uneven distribution of HyLMN in the nuclear lamina is detected in the transgenic cells compared to the controls (Fig. 6E and Suppl. Video 1): the nuclear lamina acquires a fenestrated, basket-like appearance in the transgenic cells. This abnormal structure of the nuclear lamina, however, does not affect the mitotic activity of the *Hydra* stem cells. Surprisingly, no difference in the BrDU-incorporation index is detected after 3 h and 72 h incubation in BrDU, indicating that neither the cell cycle length, nor the proportion of proliferating cells are affected (Fig. 6F, G). In accordance with these observations, clonal growth of the transgenic polyps is not affected, and the transgenic polyps have a population doubling time equal to that of the control polyps (Fig. 6H, I). No developmental abnormalities were detected in the transgenic polyps, and transgenic lines are maintained in the lab over 3 years. Together these data indicate that overexpression of HyLMN does not compromise stem cell proliferation and non-senescence in *Hydra*.

### Overexpression of a mutated HyLMN does not affect stem cell activity

**Figure 7:**
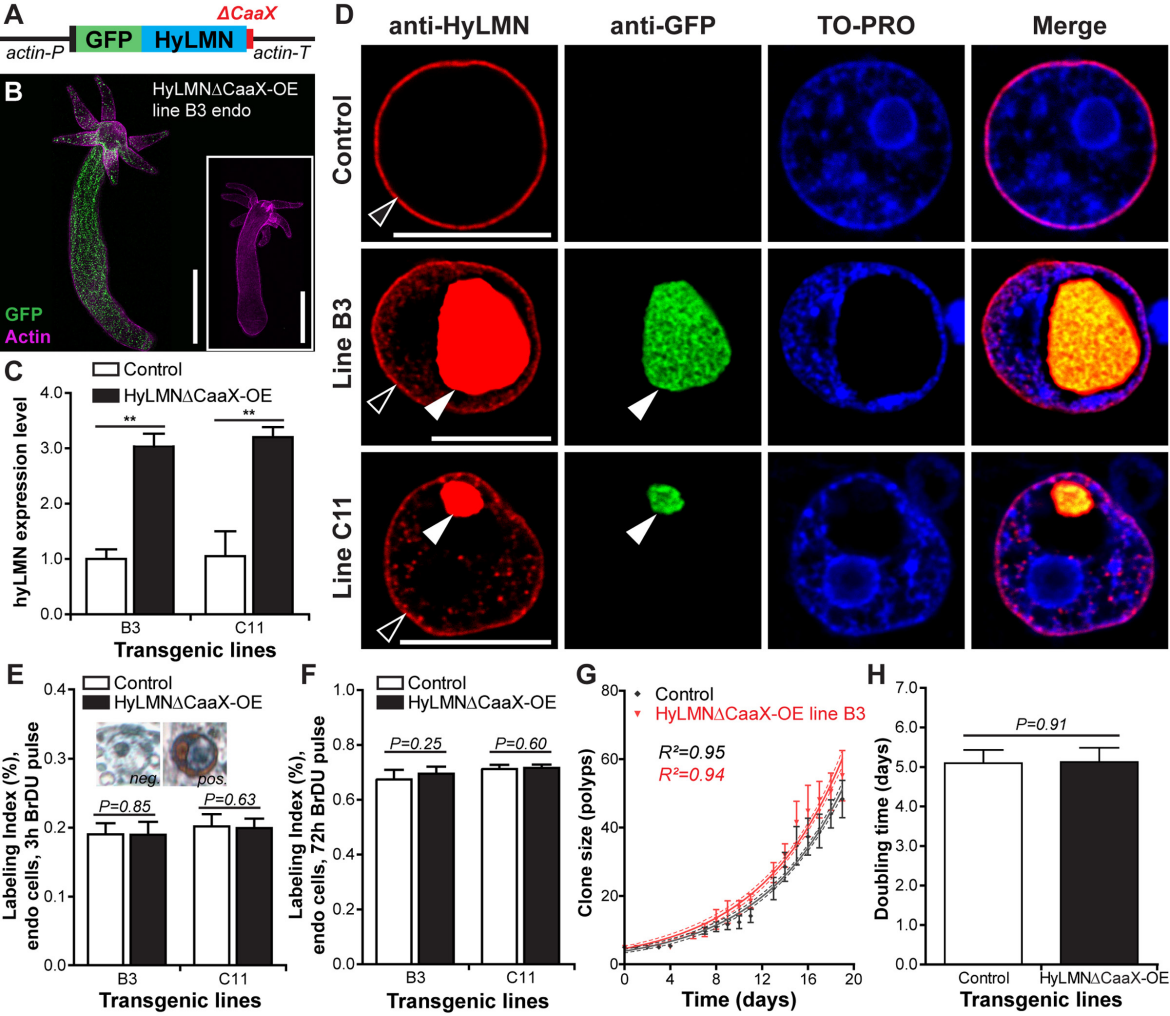
**Overexpression of HyLMN lacking CaaX-box does not affect stem cell activity.** (**A**) Construct used for overexpression of the truncated HyLMN (HyLMNΔCaaX) fused to GFP. C-terminal CaaX-motif (red) is deleted. (**B**) A representative polyp from a line B3 overexpressing HyLMNΔCaaX-GFP fusion protein in all endodermal cells, stained with anti-GFP antibodies (GFP, green) and Phalloidin (Actin, magenta). Inset - empty control polyp. Scale bar: 500 μm. (**C**) *hyLMN* mRNA expression levels in two HyLMNΔCaaX-OE lines (B3 and C11) and respective controls, analyzed by qRT-PCR (B3 *n*=3, C1 *n*=4; mean±S.D.). Asterisks indicate significant changes in expression levels (Mann-Whitney test); P values: B3 line = 0.001, C11 line = 0.003. (**D**) Aggregation of the HyLMNΔCaaX protein in the nucleoplasm of transgenic epithelial cells (white arrowhead), as evidenced by immunostaining with anti-HyLMN (red) and anti-GFP (green) antibodies. Chromatin (TO-PRO staining, blue) is displaced to the nuclei periphery. Endogenous HyLMN protein forms nuclear lamina in both, control and transgenic cells (empty arrowhead). Scale bar: 10 μm. (**E**) BrdU-labeling index of endodermal epithelial cells in the HyLMNΔCaaX-OE (B3 and C11 lines) and control polyps after 3 h exposure to BrdU (B3 *n*=10, C11 *n*=13, 494.2±99.65 cells per replicate, mean±S.D.). Inset: Transgenic cells are able to incorporate BrDU in spite of HyLMNΔCaaX aggregation. BrDU-positive (pos.) and BrDU-negative (neg.) nuclei of transgenic cells. (**F**) BrdU-labeling index of endodermal epithelial cells in the HyLMNΔCaaX-OE (B3 and C11 lines) and control polyps after 72 h exposure to BrdU (B3 *n*=10, C11 *n*=10, 470.7±29.9 cells per replicate, mean±S.D.). (**G**) Growth curves for HyLMNΔCaaX-OE line B3 and control polyps (*n*=6 replicates, each five polyps on day 0; mean±S.D., linear regression lines with 95% CI corridors and goodness of fit R^2^). (**H**) Population doubling time (mean±95% CI) for HyLMNΔCaaX-OE line B3 and control polyps derived from the plot on G.

Further we overexpressed a mutated version of HyLMN fused to GFP, where a stop-codon was inserted into the *hyLMN* coding sequence upstream from the codons for CaaX-motif (Fig. 7A), resulting in a truncated Lamin version (HyLMNΔCaaX). Two transgenic lines (B3 and C11) expressing the construct in the endodermal cells were obtained (Fig. 7B, C), and a 3-fold upregulation of *hyLMN* expression was achieved (Fig. 7C). Strikingly uneven distribution of the mutated HyLMNΔCaaX was observed in the both transgenic lines (Fig. 7D). The fusion protein is accumulated in the nucleoplasm, forms small speckles and giant granules that displace the chromatin to the nuclear periphery (Fig. 7D). As anticipated, in the absence of CaaX-motif the fusion protein is not able to be integrated in the nuclear envelope resulting in an absence of any GFP signal in the rim around the chromatin (Fig. 7D). This indicates that the nuclear lamina of the transgenic cells is made of only the endogenous non-mutated Lamin. A BrDU-incorporation analysis revealed that the transgenic cells are able to undergo cell cycle and incorporate BrDU (Fig. 7E) in spite of dramatic accumulation of Lamin in the nucleoplasm. Surprisingly, no change in proliferative activity of epithelial cells is detected after 3 h and 72 h of BrDU labeling (Fig. 7E, F). Similarly to the overexpression of a normal HyLMN (Fig. 6H, I), the clonal growth of transgenic animals with a mutated HyLMNΔCaaX is not affected (Fig. 7G), and the growth rates are equal to those of the control polyps (Fig. 7H). Taken together, our data indicate that the stem cell activity is robust against overexpression and major mislocalization of the HyLMN protein.

### Constitutive knock-down of *hyLMN* is lethal

**Figure 8:**
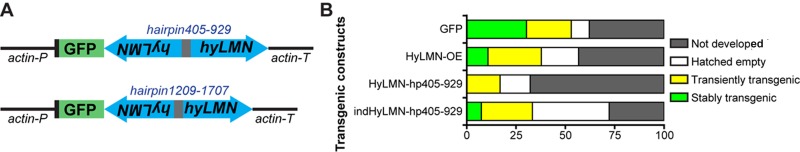
**Constitutive knock-down of *hyLMN* is lethal.** (**A**) Two shRNA constructs used for constitutive knock-down of *hyLMN* - hairpin405-929 and hairpin1209-1707. Both were driven by the actin promotor (actin-P) and flanked by actin terminator (actin-T) sequences and contained a reporter GFP sequence. (**B**) Embryos injected with the constitutive HyLMN hairpin construct (HyLMN-hp405-929, *n*=163) showed dramatically lower transgenesis efficiency compared to the embryos injected with a control construct based on the same vector lacking the hairpin cassette (GFP: *n*=66) or to the embryos injected with the HyLMN overexpression construct (HyLMN-OE: *n*=37). Efficiency of the transgenesis with the same hairpin in the inducible vector backbone (indHyLMN-hp405-929, *n*=54) was restored.

To address further the role of HyLMN in the stem cell activity, we implemented a loss-of-function approach by a gene knock-down using small hybridizing RNA (shRNA), previously shown to be very efficient in *Hydra* [9,38,39]. First, we developed a shRNA-construct (hairpin405-929, Fig. 8A) targeting the 5’-portion of *hyLMN* CDS (Suppl. Fig. 1), and cloned it under a constitutive ubiquitous actin promotor (Fig. 8A). We have injected 163 *Hydra* embryos, yet no stable transgenic line was obtained (Fig. 8B). Embryos, where any GFP signal was temporarily visible shortly after injection, either never hatched or lost the GFP signal few days after hatching. All surviving hatchlings were GFP-negative (Fig. 8B). This was in contrast to the efficiency of transgenesis with a similar plasmid backbone encoding only GFP (LigAF vector) or GFP fused to HyLMN (Fig. 6A), where about 60% of injected embryos hatch, and 30% to 50% of them stably carry a transgenic construct (Fig. 8B), consistent with the previous observations [5,39]. To exclude the off-target effects of transgenesis, we developed a second construct (hairpin1209-1707, Fig. 8A) with a shRNA-cassette against the 3’-portion of *hyLMN* CDS (Suppl. Fig. 1). Again, no stable transgenic lines were obtained with this construct. Taken together, these results indicate that the phenotype of the constitutive *hyLMN* knock-down compromises the survival of the transgenic cells or the whole embryos, and therefore, HyLMN appears indispensable for *Hydra* stem cells.

### Inducible knock-down of *hyLMN* does not affect stem cell activity

**Figure 9:**
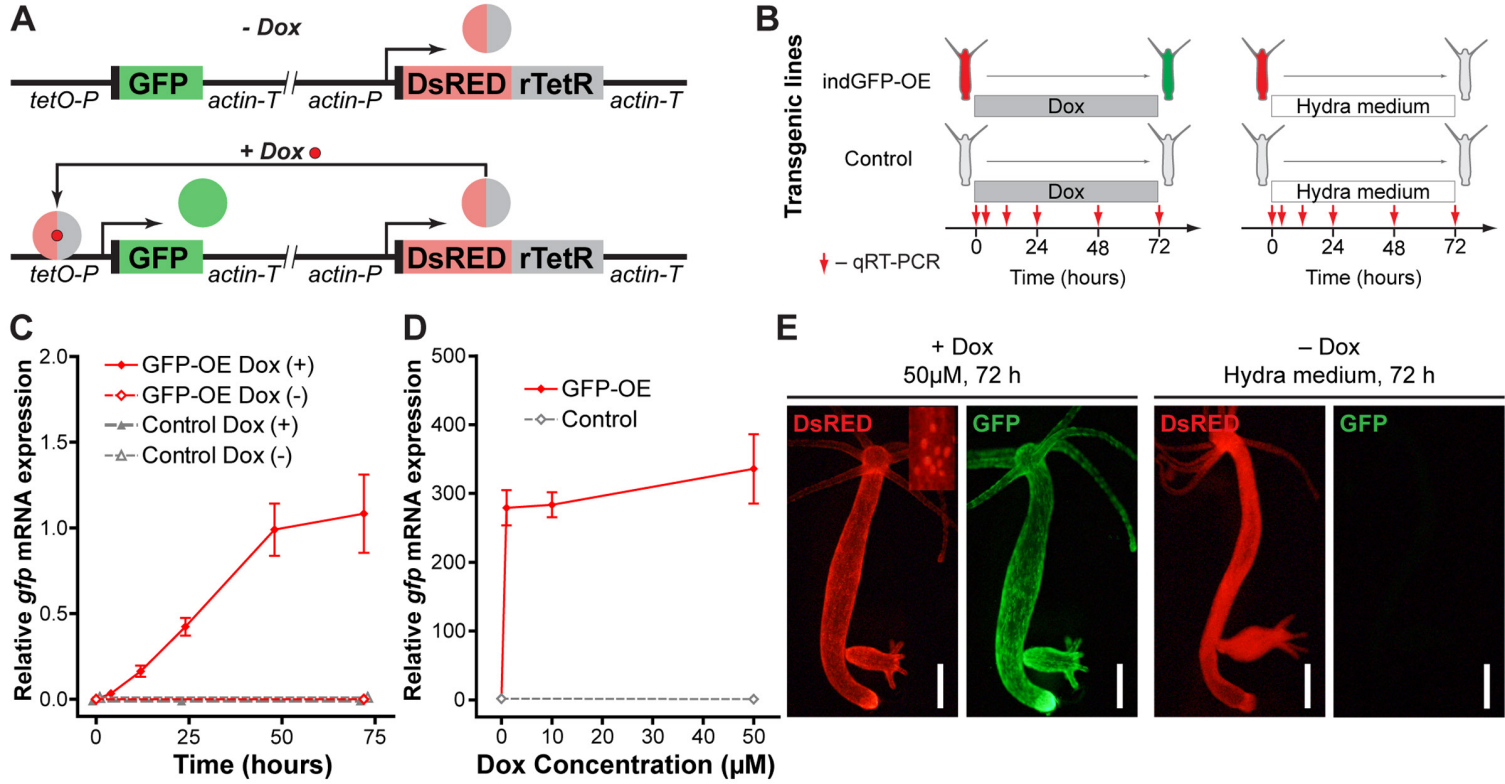
**Development and validation of the inducible gene expression system.** (**A**) Construct design and function principle of the inducible gene expression system. The gene of interest (GFP) is cloned under the control of a tetracycline-sensitive promotor (tetO-P, see Methods). Constitutive ubiquitous actin promotor (actin-P) drives expression of a tetracycline-responsive transcriptional transactivator (rTetR) fused to DsRED fluorescent protein. In the absence of doxycycline (-Dox, top), the protein is not able to bind to DNA. In the presence of doxycycline (+Dox, bottom) DsRED-rTetR fusion protein binds the tetO-P and activates expression of GFP. (**B**) Experimental setup for validation of the inducible system by qRT-PCR. Transgenic polyps containing the inducible construct (indGFP-OE) and corresponding empty controls were incubated in 10 μM doxycycline solution (Dox) or *Hydra* medium. Samples were taken at indicated time points for expression analysis by qRT-PCR. (**C**) Evaluation of the *gfp* expression level by qRT-PCR reveals time-dependent response of the transgenic indGFP-OE polyps to Dox treatment (*n*=3 for each time point, mean±S.E.M.). (**D**) Evaluation of the *gfp* expression level by qRT-PCR reveals efficient induction after 72 h treatment with Dox concentrations from 1 to 50 μM (*n*=3 for each point, mean±S.E.M.). (**E**) Life imaging of representative polyps of the indGFP-OE line treated for 72 h with doxycycline (50 μM, +Dox) or with *Hydra* medium (Dox-). DsRED fluorescence (red) is present in all epithelial cells of the polyps and localized to the nuclei (inset). Only in the presence of doxycycline (+Dox, left) GFP fluorescence (green) is detected. Scale bar: 300 μm.

**Figure 10:**
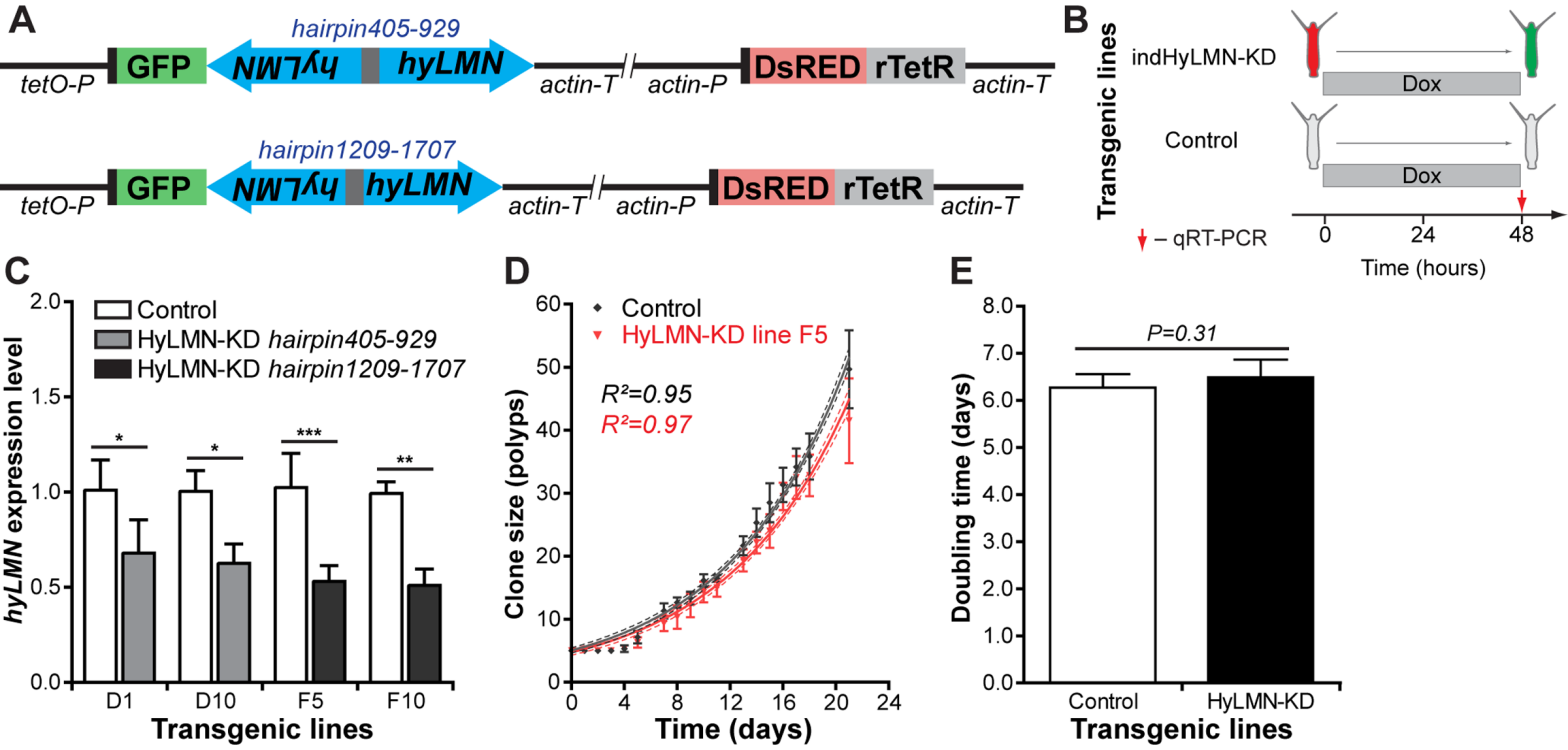
**Inducible knock-down of *hyLMN* does not compromise the growth of *Hydra*.** (**A**) Two shRNA constructs used for inducible knock-down of *hyLMN* - indHyLMN-hp405-929 and indHyLMN-hp1209-1707. (**B**) Transgenic polyps (indHyLMN-KD) and corresponding empty controls were incubated in 10 μM doxycycline (Dox) for 48 h prior to evaluation of the *hyLMN* knock-down efficiency by qRT-PCR. (**C**) *hyLMN* mRNA expression levels in two indHyLMN-KD hairpin405-929 lines (D1: *n*=4, and D10: *n*=3, mean±S.D.) and two indHyLMN-KD hairpin1209-1707 lines (F5: *n*=6, and F10: *n*=6) and in the respective controls. Asterisks indicate significant changes in expression (Mann-Whitney test); P values: D1 line = 0.039, D10 line = 0.022, F5 line < 0.001, F10 line = 0.004. (**D**) Growth curves for the indHyLMN-hp1209-1707 line F5 and control polyps (*n*=6 replicates, each five polyps on day 0; mean±S.D., linear regression lines with 95% CI corridors and goodness of fit R^2^). (**E**) Population doubling time values (mean±95% CI values) for the indHyLMN-hp1209-1707 line F5 and control polyps derived from the plot on D.

In order to overcome the lethality of the constitutively expressed constructs, we developed an inducible gene expression system based on a tetracycline-dependent transcriptional activation [40] (see Materials and Methods and Fig. 9). We further recloned the previously tested *hyLMN* shRNA-cassettes (hairpin405-929 and hairpin1209-1707) into the inducible vector (Fig. 10A). Remarkably, the efficiency of transgenesis with both constructs improved compared to constitutively active constructs. For instance, four stably transgenic lines were obtained from 54 embryos injected (4/54, 7.4% efficiency) with the inducible construct containing the hairpin405-929 (Fig. 8B). Further, we analyzed two lines containing the construct indHyLMN-hairpin405-929 (line D1 and D10) and two lines that incorporated the indHyLMN-hairpin1209-1707 construct (line F5 ad F10). In all lines, transcription of *hyLMN* gene was successfully downregulated after 48 h incubation in Dox, as determined by real-time PCR (Fig. 10B, C). Again, since only one of three stem cell lineages was transgenic in each case, the effective knock-down in the lineage affected by shRNA might be much stronger, approaching almost complete depletion of *hyLMN* mRNA. Surprisingly, in spite of the significant reduction of *hyLMN* level, Dox-induced *hyLMN* knock-down polyps showed no difference in the population growth rate compared to the corresponding control lines induced with Dox (Fig. 10D, E). This indicates that *Hydra* stem cells activity is robust against a down-regulation of *hyLMN* expression as well.

### Non-senescence is coupled to simple nuclear envelope architecture

**Figure 11:**
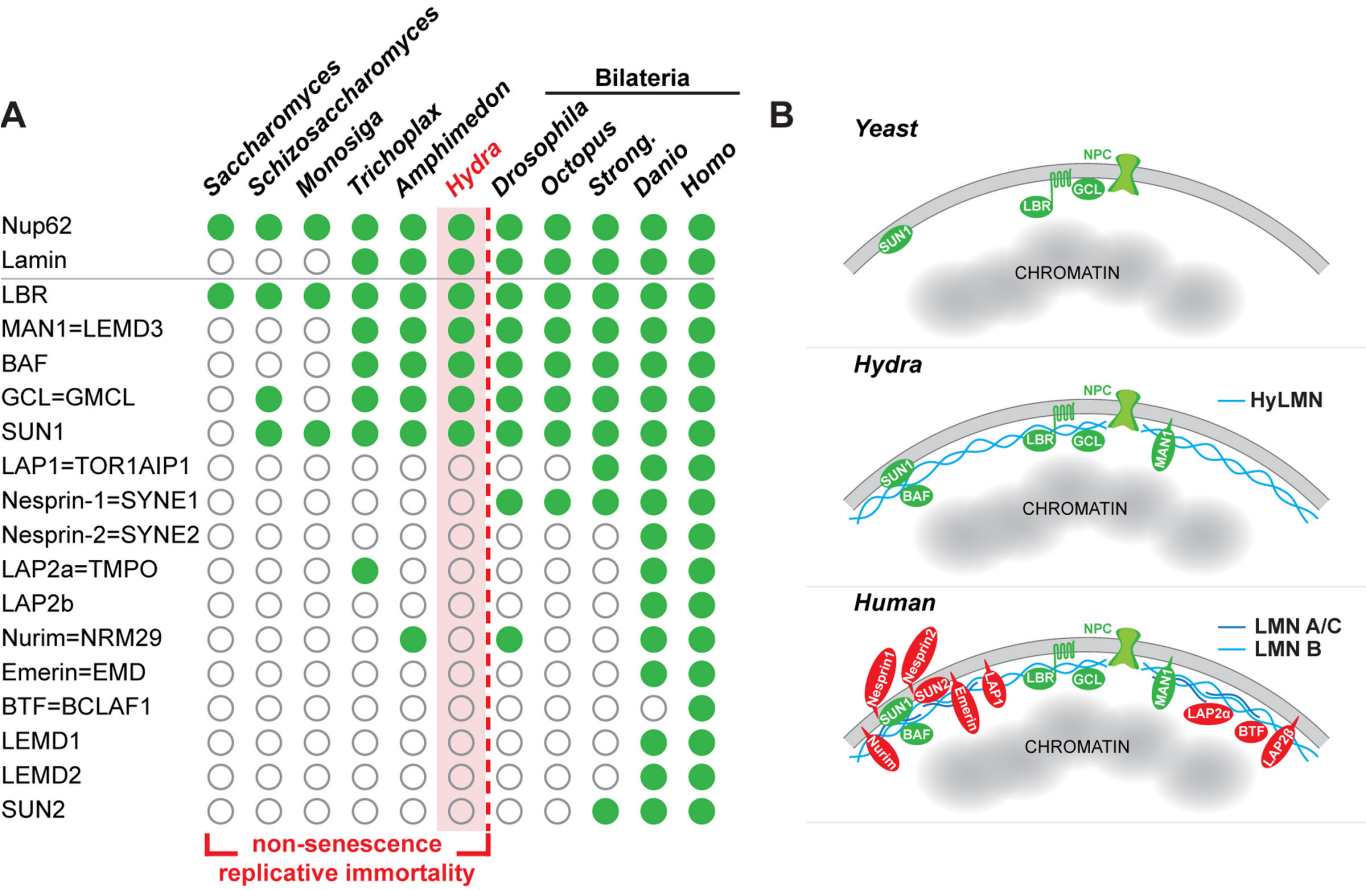
**The complexity of the nuclear lamina architecture in *Hydra* and other pre-bilaterian animals resembles that of the unicellular eukaryotes.** (**A**) Phylogenetic distribution of genes coding for Lamin and lamin-binding proteins (rows) as revealed by a BLAST-screening of genomes (columns) from unicellular and multicellular eukaryotes. Filled green circles indicate genes identified with high confidence, open circles indicate genes not found or identified with low confidence. Raw data including e-values and sequence accession numbers are presented in the Supplementary Dataset 1. Nuclear pore protein Nup62 used as a control demonstrates ubiquitous presence in eukaryotic organisms. The repertoire of genes coding for lamin-binding proteins in *Hydra* is essentially similar to that of a placozoan *Trichoplax*, sponge *Amphimedon*, and the unicellular organisms - *Sacchoromyces*, *Schizosaccharomyces* and *Monosiga*. We suggest that low complexity of the nuclear envelope in these animals enables for their non-senescence and replicative immortality. Members of the Bilateria are characterized by a rich repertoire of lamin-binding proteins and higher complexity of the nuclear envelope. Somatic cells of these animals lose replicative immortality, ultimately leading to a restricted lifespan. (**B**) A model representing increasing complexity of the nuclear envelope architecture in eukaryotes - from yeast and pre-bilaterian Metazoa, as *Hydra*, to human. Conserved proteins are in green, lamin-binding proteins that are present only in higher bilaterian animals are highlighted in red.

The robustness of the *Hydra* stem cell activity against disturbances in the HyLMN levels and in nuclear lamina structure (Fig. 6, 7, 10) contrasts with the observed high structural and functional conservation of the HyLMN protein (Fig. 2, Suppl. Fig. 1). We hypothesized, therefore, that the differences not in the structure of HyLMN itself, but in its interaction with lamin-binding proteins and with chromatin might account for the unique resistance of *Hydra* to Lamin disturbances. To test this hypothesis, we compared the presence of genes coding for lamin-binding proteins in *Hydra* and a sea anemone *Nemtostella,* the unicellular eukaryotes *Dictyostelium*, *Schizosaccharomyces*, *Saccharomyces* and *Monosiga*, a sponge *Amphimedon*, a placozoan *Trichoplax*, and five bilaterian animals (*Drosophila*, *Octopus*, *Strongylocentrotus*, *Danio*, *Homo*). Interestingly, from 16 lamin-binding proteins coded in the genomes of mammals, only five are found in *Hydra* (Fig. 11, Suppl. Dataset 1). Few genes coding for lamin-binding proteins are also found in another cnidarian, *Nematostella*, indicating that this limited repertoire of genes coding for lamin-binding proteins is rather typical for cnidarians and is not an artifact of poor genome sequencing and annotation. Similarly, few genes coding for lamin-binding proteins are present in the genomes of the sponge, placozoan, and in the unicellular organisms (Fig. 11, Suppl. Dataset 1). This suggests that the cnidarians retain a relatively low complexity of the nuclear envelope, made up of a single Lamin and few lamin-binding proteins. The architecture of the nuclear envelope in bilaterian animals, especially in Deuterostomia, appears strikingly different and more complex.

## DISCUSSION

A vast diversity of ageing patterns and lifespans has been reported across the animal kingdom, from fast-ageing short-living species as mayflies and turquoise killifish to species with a remarkable longevity and a negligible senescence - as clams and *Hydra* [4,41–43]. Studying age-related genes and genetic pathways across distant animal phyla, and especially in the species at the extremes of the lifespan range, may be instrumental in uncovering fundamental principles of the aging and lifespan control. It allows identifying mechanisms shared between the distant phyla (“public”) and those specific to certain evolutionary lineages (“private”) [44]. Extreme longevity of cnidarians makes these animals excellent models for getting insights into the molecular basics of ageing and lifespan control, and to understand the evolution of extended lifespans [45,46]. In the recent decade research on the freshwater polyp *Hydra* revealed several genes and cellular processes enabling *Hydra* to decouple the aging process from its life history [9,47]. Here, by gain- and loss-of-function experiments we show that although the single Lamin protein is indispensable for *Hydra,* similarly to B-type Lamins in other model organisms [27,48–50], *Hydra* cells appear invulnerable to the changes in Lamin expression level and localization. Since *Hydra* has only one *lamin* gene, any compensatory effects from other *lamin* homologs can be excluded. In bilaterian animals, on the contrary, in spite of the existence of multiple *lamin* genes (two in *Drosophila*, three to four in vertebrates [35,37]) and a potential redundancy in their function, the nuclear lamina appears to be extremely vulnerable. Overexpression of Lamin A or B1 [51,52], overexpression of the truncated Lamins lacking CaaX-box [27], silencing of the *lamin A/C* or *B* expression [28,53,54], as well as inactivating mutations in the *lamin A* gene [55] impair replicative potential of cells and decrease the animal’s lifespan. Remarkably, all the models used in these studies (fly, flatworm, frog, mice and human) belong to the Bilateria clade and display senescence. *Hydra* belongs to the sister group of Bilateria – the Cnidaria phylum and demonstrates continuous growth and non-senescence, that are characteristic for other pre-bilaterian animals, such as corals and sponges, as well [45,46]. Our observations suggest that non-senescence of pre-bilaterians requires special nuclear anatomy.

We hypothesize further, that fundamental differences in the repertoire of proteins interacting with Lamins and mediating their functions, known as lamin-binding proteins, might account for the extraordinary independence of *Hydra* stem-cell activity from the nuclear envelope architecture. Our analysis revealed that, in contrast to bilaterian organisms, and in particular to vertebrates and humans, the repertoire of genes coding for putative lamin-binding proteins is very much restricted in *Hydra* and other cnidarians, sponges and placozoans (Fig. 11A). The nuclear lamina complexity in early-branching metazoans is strikingly similar to that of the unicellular organisms, where only few lamin-binding proteins are present [56]. Interestingly, similarly to the pre-bilaterians, unicellular organisms are known for their replicative immortality [57–59]. We suggest that the fundamental difference in the nuclear envelope architecture and function may explain why in pre-bilaterian animals (*e.g*. sponges and cnidarians) stem cells display an everlasting activity, enabling non-senescence and extreme longevity of these animals (Fig. 11A, B). In Bilateria, on the contrast, the increasing complexity of the lamina (increasing number of Lamin proteins, expanding diversity of lamin-binding proteins) may underlie their nuclei “fragility” and strong dependence of the replicative function on the lamina. This, in turn, predisposes cells to a replicative senescence and ultimately - to a restricted lifespan.

Two observations support this scenario. First, the only cell type in vertebrate animals that is independent on the Lamin expression and localization is the embryonic stem cells. In these cells, the gene coding for Lamin A/C is not expressed, while fluctuations in expression of B-type Lamins do not cause any deleterious effects [60,61]. Importantly, embryonic stem cells, in contrast to other somatic cells, are characterized by a replicative immortality [43]. Second, our model suggests an essential role of the lamin-binding proteins in controlling the cellular senescence. A plethora of diseases, phenotypically similar to those, caused by mutations in the *lamin* genes, are induced by mutations in genes coding for lamin-binding proteins, such as LBR, EMD, MAN1, LAP2 [62].

Our work has shown that the non-senescent, long-lived organisms such as *Hydra* can provide important insights into the mechanisms of aging and longevity. Understanding these processes can have significant implications for managing genetic disorders in human such as laminopathies, and for developing approaches of the “healthspan” extension.

## MATERIALS AND METHODS

### Animals and culture conditions

Experiments were carried out using *Hydra vulgaris* strain AEP. Animals were maintained under constant conditions including the culture medium, food, and temperature (18°C) according to standard procedures [63].

### Generation of transgenic *Hydra* strains

To overexpress HyLMN protein, the coding region of *hyLMN* was cloned into the LigAF vector [5] in-frame and downstream from the sequence of an enhanced green fluorescent protein (eGFP). The transgenic cassette was therefore driven by the actin promoter sequence and flanked by the actin terminator from the 3’-end. To overexpress a truncated version of HyLMN lacking CaaX-motif (HyLMNΔCaaX), a stop-codon was introduced into the *hyLMN* CDS upstream adjacent to the codons coding for CaaX-box. To induce shRNA-mediated knock-down of *hyLMN*, two hairpin cassettes were designed based on previously reported principles [38]. Briefly, each hairpin contained a sequence of about 500 bp in sense and antisense orientation, separated by a short (300 bp) non-complementary spacer region (Fig. 8A). After the assembly, the hairpin cassette was cloned behind the *eGFP* CDS into the LigAF vector [5] (Fig. 8A), or into the backbone of the inducible system (indGFP-OE, Fig. 9A, 10A). All constructs were propagated in the *E. coli* DH5-alpha strain and microinjected into fertilized embryos of *H. vulgaris* strain AEP as previously described [5]. To compare the transgenesis efficiency, LigAF vector containing only GFP cassette was injected. Initial founder mosaic transgenic animals were screened and enriched for transgenic cells until all cells of a given lineage were transgenic. For each transgenic line a corresponding control line (so called “empty”) was obtained by screening and depleting an initially mosaic polyp until no transgenic cells were detected. Transgenic and “empty” polyps were expanded into mass cultures by clonal propagation by budding and used in further experiments.

### Generation of anti-HyLMN antibodies

A peptide corresponding to the C-terminal portion of HyLMN (amino acids 390-536, Suppl. Fig. 1) was recombinantly expressed as a C-terminally His-tagged fusion protein in pET21-a(+) vector (Novagen, Darmstadt, Germany) in *E. coli* Rosetta(DE3)pLysS and purified on Ni-NTA-Agarose (Qiagen, Hilden, Germany). The antigen was used to immunize one rabbit and one guinea-pig (SEQLAB, Göttingen). Polyclonal antibodies were affinity purified on the antigen coupled to a HiTrap NHS activated column (GE Healthcare, München) and concentrated using Centriplus YM 50 Centricons (Millipore, Melbourne, Australia) up to 1 mg/ml.

### Immunohistochemistry on whole animals and macerates

Immunohistochemical detection in whole mount *Hydra* preparations was performed as described previously [64]. Briefly, polyps were relaxed in 2% urethane in *Hydra* medium, fixed in 4% PFA in *Hydra* medium, washed with 0.1% Tween in PBS, and permeabilized with 0.5% Triton X-100 in PBS, incubated in blocking solution (1% BSA, 0.1% Tween in PBS) for 1 h and incubated further with primary antibodies diluted in blocking solution at 4°C. Custom polyclonal rabbit and guinea-pig antisera against a synthetic peptide of HyLMN were used at 1:200 dilutions. Polyclonal rabbit anti-GFP antibodies (Millipore) and monoclonal mouse anti-GFP antibodies (Roche, Mannheim, Germany) were diluted 1:500. AlexaFluor546-conjugated donkey-anti-rabbit and goat-anti-mouse, AlexaFluor488-conjugated goat-anti-rabbit and donkey-anti-mouse, and AlexaFluor555-conjugated goat-anti-guinea pig antibodies (all Invitrogen, Eugene, OR, USA), were all diluted to 4 μg/ml in blocking buffer and incubations were done for 1 h at room temperature. Rhodamin-phalloidin (Sigma, Steinheim, Germany) and TO-PRO3-iodide-AlexFluor633 (Invitrogen, Eugene, OR, USA) counterstaining was conducted as described previously [65]. For labeling of the cells from dissociated *Hydra* polyps, macerations were prepared as previously described [66]. Slides were dried for at least 3 h and labeling was carried out using the same steps described for the whole mount immunostaining. Confocal laser scanning microscopy was done using a TCS SP1 laser scanning confocal microscope (Leica, Wetzlar, Germany).

### Transmission electron microscopy and immunogold labeling

To reveal the structure of the nuclear envelope in *Hydra* cells, we performed electron microscopy analysis as previously described [67]. Briefly, the polyps were fixed with 2,5% glutaraldehyde solution, treated further with 2% osmium tetroxide and embedded into Epon 812. Further transmission electron microscopy analysis revealed good preservation of the cellular structures (Fig. 4H, I). However, effort to localize further the HyLMN using specific antibodies fail, most likely due to epitope damage caused by the glutaraldehyde fixation. Therefore, we used further the “pre-embedding protocol” [67] with immunogold labeling. Briefly, cryosections of *Hydra* polyps were fixed with 2% formaldehyde and treated consequently with primary anti-HyLMN antibodies and secondary anti-rabbit antibodies conjugated to 6 nm gold particles. The samples were further fixed with 2,5% glutaraldehyde solution, treated with 2% osmium tetroxide and embedded into Epon 812. This procedure resulted in sufficient epitope preservation, and a deposition of gold particles was specifically restricted to the inner surface of the nuclear envelope (Fig. 4J). In this case, however, the morphology of the cells was impaired, and therefore, the method was not used further for transgenic phenotype characterization.

### Western blotting

Protein extracts were obtained by homogenizing 10 *Hydra* polyps on ice in 2× Sample buffer supplemented with 200 mM dithiothreitol, denatured by boiling for 5 min, resolved in a 10% SDS–PAGE and transferred onto the Roti-PVDF membrane (Roth GMbH, Karlsruhe, Germany). The membrane was incubated in the rabbit anti-HyLMN (0.3 μg/ml in 4% skimmed milk, 1% BSA supplemented blocking solution) overnight at 4°C. Immune complexes were detected by the peroxidase-labeled goat-anti-rabbit polyclonal antibodies (Millipore) and developed using NBT/BCIP substrate (Roche Diagnostics, Mannheim, Germany).

### *In situ* hybridization

Expression pattern of the *hyLMN* gene was detected in the whole mount *Hydra* preparations by *in situ* hybridization with an anti-sense digoxigenin (DIG) -labeled RNA probe [68]. DIG-labeled sense-probe was used as a control. Signal was developed using anti-DIG antibodies conjugated to alkaline phosphatase (1:2000, Roche) and NBT/BCIP staining solution (Roche). Images of the *in situ* preparations were collected on a Zeiss Axioscope microscope with Axiocam camera.

### Proliferation assay

BrDU labeling was used to analyze the stem cells proliferation in *Hydra*. Polyps were incubated in 5 mM 5-bromo-2′-deoxyuridine (BrdU, Sigma, Steinheim, Germany) solution for 3 h (pulse-labeling) or in 2 mM BrdU for 72 h (continuous labeling), with the solution being additionally injected into the polyp gastric cavity every 12 h. After 3 h or 72 h incubation two polyps were pooled for each replicate and macerated into a single-cell suspension, as described previously [66]. Immunodetection of BrdU on slides was done as described previously [9]. Labeling index was assessed as a percentage of the cells labelled with BrDU from the total number of cells of a certain type.

### Growth rate assay

To assess the growth rate, clonal lines were established by placing 5 founder polyps per well into a 6-well plate. The polyps were fed daily *ad libitum*. The number of the clonal progeny polyps propagated by budding within each clonal line was counted daily for a period of four weeks.

### Quantitative real-time PCR gene expression analysis

To estimate the expression level of *hyLMN* and *gfp*, we performed quantitative real-time PCR. Total RNA was extracted from transgenic polyps and converted into the cDNA as previously described [68]. Real-time PCR was performed using GoTaq qPCR Master Mix (Promega, Madison, USA) and oligonucleotide primers specifically designed to *hyLMN* and *gfp*, as well as *ef1a* (translation elongation factor 1 alpha) and *actin* genes as equilibration references (Suppl. Table 1). The data were collected by ABI 7300 Real-Time PCR System (Applied Biosystems, Foster City, USA) and analyzed by the conventional ΔΔCt method.

### Development of an inducible gene expression system

To develop an inducible gene expression system, we introduced the following modifications into the standard LigAF vector (Fig. 9A). The actin promotor in front of the GFP coding sequence was substituted by a minimal CMV promotor (169 bp, with an internal Kozak sequence) fused to a 7 times repeated tetracycline-sensitive operator sequence (tetO, 7x19 bp separated by linkers of 16-17 bp, 250 bp in total). Downstream from the GFP coding sequence and actin terminator a second cassette was introduced, made of an actin promotor (1420 bp) and actin terminator (711 bp) sequences flanking a coding sequence for a modified tetracycline-responsive transcriptional transactivator (rTetR) fused to DsRED (Fig. 9A). The nucleotide sequence coding for the rTetrR_rtTA-M2 variant [40] (207 amino acids) was fused to the sequences coding for a nuclear localization signal (PKKKKKRAKKDP) and for three F-type VP16 activation domains [69,70] (each 39 amino acids long). All nucleotide sequences were optimized for codon usage of *Hydra*. The principle of inducible system function is presented on Fig. 9A. The plasmid and its sequence are available upon request.

First, we validated the system by expressing the fluorescent protein GFP alone (Fig. 9). In the absence of the inducer only the red signal (DsRED) concentrated in the nuclei was detected in the transgenic polyps. Twenty-four hours after addition of Dox visible green signal appeared, and became more intensive in the following 48 hours (Fig. 9E). In agreement with that, quantification of the *gfp* expression by qRT-PCR revealed a time-dependent response (Fig. 9C). Concentrations of Dox as low as 1 μM efficiently induced the expression of *gfp* mRNA (Fig. 9D). Upon withdrawal of the Dox, the GFP signal became weaker in the next 2 days, and completely disappeared few weeks later (data not shown), indicating that the expression induction is reversible. No developmental abnormalities or growth disturbances were observed in the transgenic lines. Moreover, the same line can be induced multiple times (data not shown). Taken together, these results indicate successful establishment of the inducible gene-expression system, which can be further used for tightly controlled gene manipulation in *Hydra*.

### Expression in the heterologous system

A fibroblast-like COS-7 (ATTC CRL1651) cell line derived from monkey kidney was used for heterologous expression of HyLMN. A full-length coding sequence of *hyLMN* was cloned into the pCMV-Myc vector (Clontech) to result in an N-terminally Myc-tagged fusion protein. To overexpress a truncated version of HyLMN (Myc-HyLMNΔCaaX), a stop-codon was introduced before the nucleotides coding for CaaX-motif. COS-7 cells were cultured in DMEM-Medium (Sigma) at 37°C und 5% CO_2,_ transfected using Magnet Assisted Transfection (IBA Lifesciences, Goettingen, Germany), and analyzed 24 h after the transformation using immunofluorescence. Briefly, the cells grown on cover slips were fixed in 1% formaldehyde in PBS and permeabilized by 0.1% Triton supplemented PBS, and stained for 30 min with rabbit polyclonal anti-Myc antibody (1:200, Millipore) in PBT. Further, the slides were stained with AlexaFluor488-conjugated goat-anti-rabbit antibodies (1:100, Invitrogen), counter-stained with Hoechst 33258 (Sigma) and scanned using TCS-SP2 laser scanning confocal microscope (Leica).

### Phylogenetic analysis of HyLMN protein

Phylogenetic analysis of HyLMN was based on its predicted full-length amino acid sequence. ClustalW [71] with default parameters was used to align sequences of annotated Lamin homologues from the following species: *Homo sapiens* (accession numbers NP_733821, AAH12295, AAH06551), *Gallus gallus* (NP_990618, NP_990617, NP_990616), *Xenopus laevis* (NP_001095210, NP_001080053, AAC31544), *Danio rerio* (CAB58234, CAB41015), *Strongylocentrotus purpuratus* (NP_999665), *Drosophila melanogaster* (CAA53480), *Acropora digitifera* (XP_015765214), *Nematostella vectensis* (XP_001629288), *Hydra vulgaris* strain AEP (this study, MG763927), *Trichoplax adhaerens* [72], *Amphimedon queenslandica* (XP_011404056), and *Monosiga brevicollis* [56]. The evolutionary history was inferred by using the maximum likelihood method [73], and the tree with the highest log likelihood (-9596.5780) was selected. The percentage of the trees in which the associated taxa clustered together is shown next to the branches. Maximum-likelihood bootstrap values were calculated based on 1000 replicates. Neighbor-Joining method, a JTT model and a discrete Gamma distribution were implemented, and the most parsimonious tree was generated using the MEGA6 [74].

### Phylogenetic distribution of genes coding for lamin-binding proteins

To analyze the presence of the genes coding for lamin-binding proteins in diverse taxa of eukaryotes, we performed a BLAST search [75] using 16 lamin-binding proteins from *Homo* against nucleotide databases (tblastn) of five bilaterian animals (*Drosophila*, *Octopus*, *Strongylocentrotus*, *Danio*, *Homo*), the cnidarians *Hydra* and *Nemtostella*, a sponge *Amphimedon*, a placozoan *Trichoplax*, and unicellular eukaryotes *Dictyostelium*, *Schizosaccharomyces*, *Saccharomyces* and *Monosiga.* As a control we used sequence of the highly conserved nuclear pore protein Nup62, known to be present in virtually all eukaryotes [76]. Matches with expectation e-value <E-05 were considered as signs of homolog presence, however were verified by manual domain composition analysis. Best hit sequences that demonstrated low similarity (e-value >E-05) and/or that retrieved members of some other gene families, not relevant to lamin-bining proteins, upon reciprocal BLAST using UniProt, were discounted. Accession numbers of the seed sequences and best blast-hits with corresponding values are presented in the Suppl. Dataset 1.

## Supplementary Material

Supplementary File

Supplementary Video 1

Supplementary Dataset 1
