# Supplementary material for: Non-senescent Hydra tolerates severe disturbances in the nuclear lamina
Source: Aging (Albany NY). 2018 May 10;10(5):951–72. doi: 10.18632/aging.101440 (PMC5990382; doi:10.18632/aging.101440)
Supplement: Supplementary File [file aging-10-101440-s001.pdf]

SUPPLEMENTARY MATERIALS

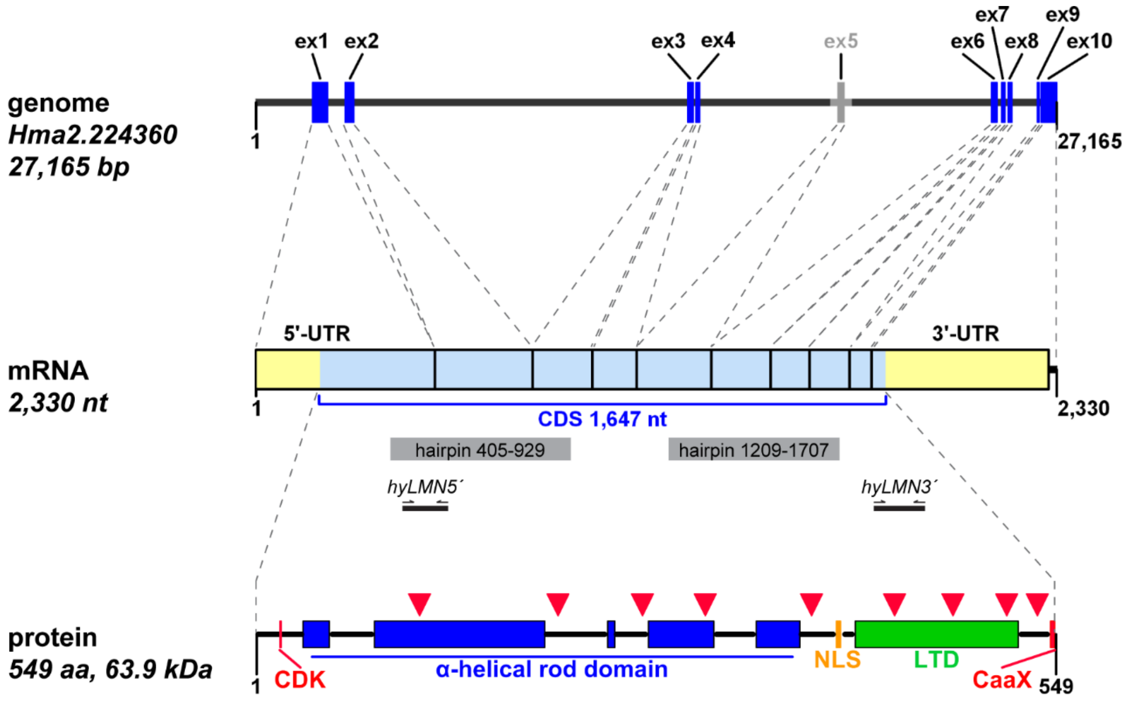

Supplementary Figure 1. *HyLMN* gene and its protein product have a highly conserved structure. A single *lamin* gene, *hyLMN*, is present in the genome of *Hydra*, represented by the gene model Hma2.224360, that spans 27,165 bp. Comparison of the genomic sequence to the cloned cDNA sequence allows to unambiguously identify nine putative exons, separated by introns of variable length. One putative exon (ex5, gray) with short adjacent intron sequences cannot be clearly located due to poor quality of the genomic sequence. The *hyLMN* transcript is 2,330 nt long and comprises a short 5'-UTR, a coding sequence of 1,647 bp, and a long 3'-UTR. The positions of two fragments amplified in real-time PCR are highlighted. Two shRNA-constructs (hairpin405-1207 and hairpin1209-1707) were created, targeting two adjacent portion of the *hyLMN* coding sequence. The CDS codes for a peptide of 549 amino acids. Intron-exon junctions (red triangles) follow a conserved pattern typical for *lamin* genes of vertebrate animals. HyLMN contains all hallmarks found in the B-type Lamins of vertebrate animals[34]: the short N-terminal domain contains a putative motif for phosphorylation by cyclin-dependent kinases (CDK, red) and is followed by an alfa-helical rod domain made up of several coiled-coil segments (blue). The C-terminal portion contains a putative nuclear localization signal (NLS, orange), immunoglobulin-like lamin terminal domain (LTD, green) and a C-terminal CaaX-like motif (CaaX, red).

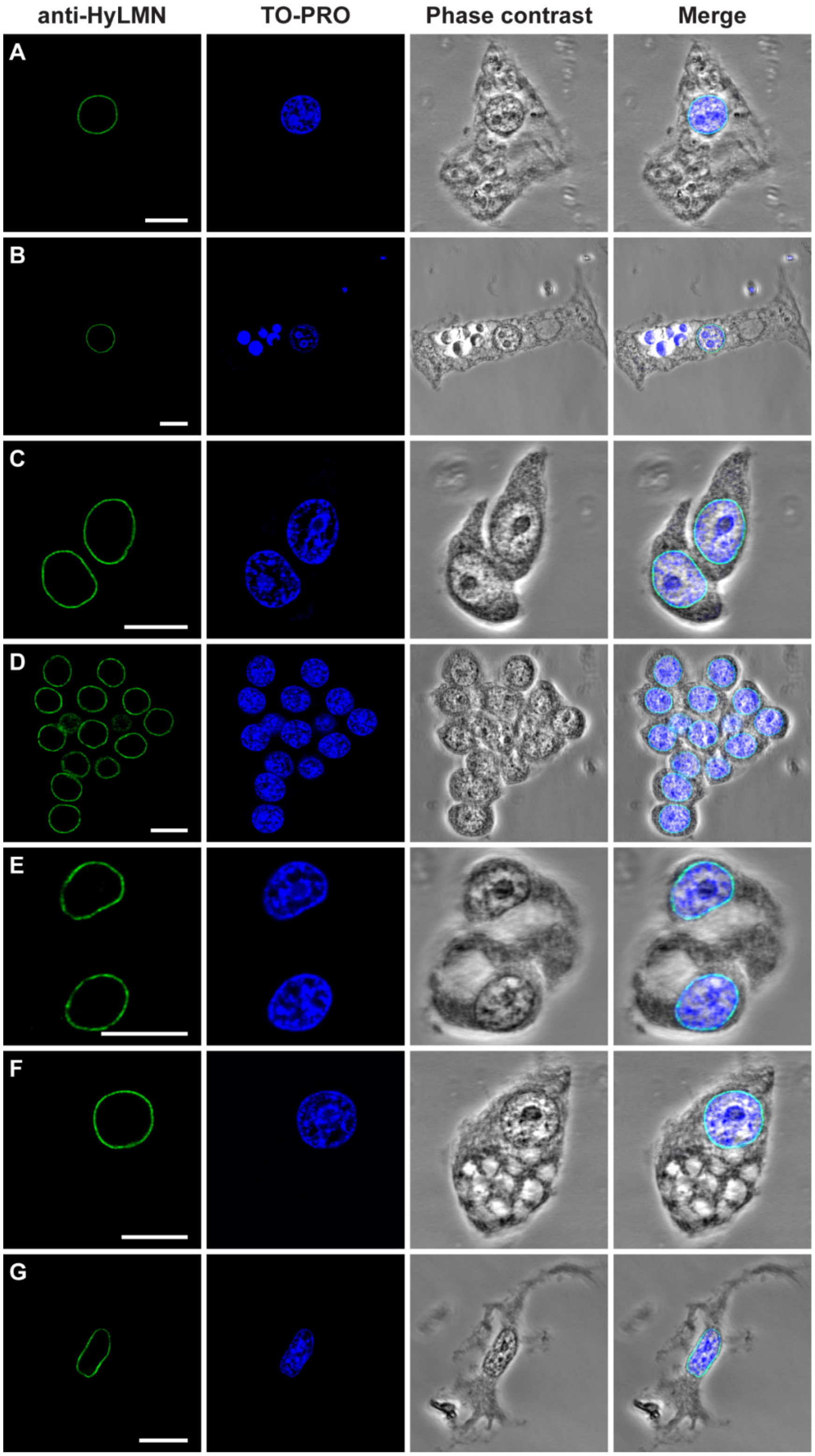

Supplementary Figure 2. HyLMN is present in the nuclei of all cell types in *Hydra*. Immunofluorescent detection of HyLMN protein on macerated cells reveals that HyLMN is present in the nuclei of stem cells and in their differentiated progeny. HyLMN forms a thin rim on the nuclear periphery – the nuclear lamina. (**A**) Ectodermal epithelial cell. (**B**) Endodermal epithelial cell. (**C**) Pair of interstitial stem cells. (**D**) Cluster of germline precursor cells. (**E**) Two differentiating nematocytes, each with a conspicuous vesicle - a growing nematocyst. (**F**) Differentiated gland cell with prominent secretory vesicles in the cytoplasm. (**G**) Differentiated ganglion neuron. Immunodetection of lamin (anti-HyLMN, green), DNA (TO-PRO, blue), phase contrast and merged image. Scale bar: 10 µm.

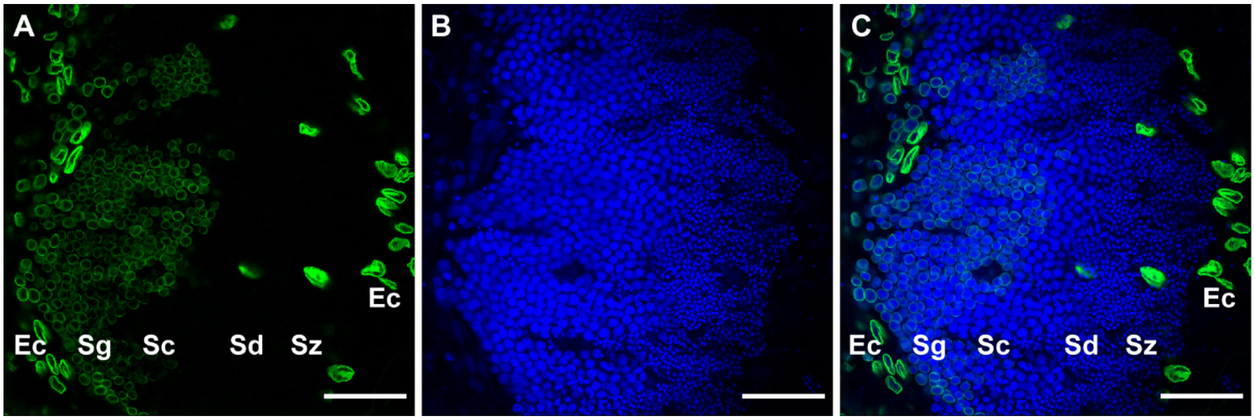

Supplementary Figure 3. *HyLMN* is strongly expressed in the proliferating germline stem cells, but absent from the differentiated gametes. (**A–C**) Immunostaining with anti-HyLMN antibodies (green) demonstrates a progressive loss of HyLMN protein by differentiating male germline cells. While mitotically dividing spermatogonia (Sg) at the basis of a testis reveal strong signal in their nuclear envelopes, in later spermatocytes (Sc), spermatids (Sd) and spermatozoa (Sz) no HyLMN can be detected. Nuclei of ectodermal epithelial cells (Ep) are visible in the basal and distal part of the testis. DNA is stained with TO-PRO3 (blue). Scale bar: 50 µm.

Supplementary Table 1. Sequences of oligonucleotide primers used to amplify gene products in real-time PCR.

PCR product	Target gene	Genbank Accession #	Forward primer 5' -> 3'	Reverse primer 5' -> 3'
act	*actin*	XM_002154426	gaatcagctggtatccatgaaac	aacattgtcgtaccacctgatag
ef1	*elongation factor 1*	JX118844	gcagtactggtgagtttgaag	cttcgctgtatggtggttcag
gfp	*green fluorescent protein*	DQ369740	gagagggtgaaggtgatgca	agtcatgccgtttcatatgatctg
hyLMN5'	*hyLMN*	MG763927	gagacttcttgacgagactgc	actcttcttcaatgcgacttcg
hyLMN3'	*hyLMN*	MG763927	ggaggcgagcgtgaatcg	cttgacaactcgaacgactgc

Supplementary Video 1. Overexpression of HyLMN results in an uneven distribution of Lamin. Immunostaining with anti-HyLMN and anti-GFP antibodies and confocal imaging (animated stack of 30 confocal slices) reveal uneven distribution of HyLMN in the nuclear lamina of the transgenic epithelial cell (HyLMN-OE) compared to the control cell. The Lamin signal (red) becomes stronger, GFP signal (green) appears, and the nuclear lamina acquires a fenestrated aspect. DNA is stained with TO-PRO (blue). Scale bar: 10 µm.

Supplementary Dataset 1. The repertoire of genes coding for lamin-binding proteins is restricted in non-senescent organisms. Phylogenetic distribution of 16 genes coding for lamin-binding proteins as revealed by a BLAST-screening of nucleotide sequence databases of 13 unicellular and multicellular organisms.
